# The Gut Microbiome in HIV Pathogenesis: Interconnections Between Dysbiosis, Immune Dysfunction, and Viral Persistence

**DOI:** 10.3390/ijms27114830

**Published:** 2026-05-27

**Authors:** Hossein Mardnaybin, Mehmet Demirci, Hayriye Kirkoyun Uysal

**Affiliations:** 1 Department of Medical Microbiology, Institute of Health Sciences, Istanbul University, Istanbul 34093, Türkiye; hossein.mardnaybin@gmail.com; 2 Department of Medical Microbiology, Istanbul Faculty of Medicine, Istanbul University, Istanbul 34093, Türkiye; hkirkoyun@yahoo.com; 3 Department of Medical Microbiology, Faculty of Medicine, Kirklareli University, Kirklareli 39100, Türkiye

**Keywords:** HIV, gut microbiome, dysbiosis, immune activation, microbial translocation, viral reservoirs, probiotics

## Abstract

The human gut microbiome is essential for immune regulation and mucosal homeostasis, functions that are profoundly disrupted during HIV infection. Early viral replication in the gut-associated lymphoid tissue (GALT) triggers a self-reinforcing cycle of CD4^+^ T-cell depletion, epithelial barrier breakdown, and increased microbial translocation. This persistent immune activation continues even under effective antiretroviral therapy (ART). A growing body of evidence indicates that HIV infection is consistently associated with alterations in gut microbial communities. This dysbiosis is typically characterized by fewer beneficial butyrate-producing commensal bacteria and an enrichment of pro-inflammatory microbial taxa. It also involves disturbances in key microbial metabolites, including short-chain fatty acids (SCFAs) and tryptophan catabolites. Such changes not only exacerbate systemic inflammation but may also contribute to incomplete immune reconstitution and the persistence of latent viral reservoirs despite long-term ART. In this review, we summarize current knowledge of microbiome–HIV interactions, with particular emphasis on the mechanisms through which gut dysbiosis contributes to immune dysfunction and viral persistence. We discuss recent advances in multi-omics technologies, as well as experimental systems such as gnotobiotic and humanized mouse models and intestinal organoid platforms that are helping to elucidate these complex interactions. Furthermore, we evaluate emerging microbiome-targeted interventions—including probiotics, prebiotics, fecal microbiota transplantation, and engineered bacterial therapeutics—and consider their potential role as adjunctive strategies in HIV treatment and cure research. By integrating microbiological, immunological, and clinical perspectives, this review highlights key knowledge gaps and outlines future research directions aimed at harnessing the gut microbiome as a novel therapeutic avenue in HIV management and eradication.

## 1. Introduction

Human immunodeficiency virus (HIV) infection continues to pose a significant global health challenge, with more than 39 million people currently living with the virus worldwide [1]. The development and widespread use of combination antiretroviral therapy (ART) has dramatically reduced HIV-related morbidity and mortality, transforming HIV infection from a fatal disease into a chronic, manageable condition. Nevertheless, ART does not eliminate the virus. Latent viral reservoirs persist in infected individuals. Furthermore, chronic immune activation and inflammation remain major drivers of long-term complications, including cardiovascular disease, neurocognitive impairment, and features of accelerated aging in people living with HIV (PLWH) [2,3,4,5]. Understanding the mechanisms underlying this persistent immune dysregulation has therefore become a central focus of contemporary HIV research.

One factor receiving increasing attention is the gut microbiome. The human gastrointestinal tract harbors trillions of microorganisms that play essential roles in nutrient metabolism, immune system development, and the maintenance of mucosal homeostasis [6]. Disruptions in microbial composition and function—collectively referred to as dysbiosis—have been linked to numerous diseases, including inflammatory bowel disease, metabolic disorders, and cancer [7]. In the context of HIV infection, growing evidence suggests that microbial dysbiosis both arises from and contributes to immune dysfunction [8].

The gastrointestinal tract is also a key site of HIV pathogenesis [9]. Within days of infection, HIV preferentially targets the gut-associated lymphoid tissue (GALT), resulting in extensive depletion of mucosal CD4^+^ T cells [10]. In particular, Th17 and Th22 subsets—critical for maintaining epithelial barrier integrity and mucosal immune defense—are disproportionately affected during early infection [9]. As these protective immune populations are lost, the intestinal barrier becomes compromised. This damage facilitates microbial translocation, allowing bacterial components such as lipopolysaccharide (LPS) and flagellin to leak from the gut lumen into systemic circulation. The resulting activation of innate immune pathways fuels chronic inflammation and contributes to a self-reinforcing cycle of immune activation, tissue injury, and viral persistence [11,12].

A growing body of research has demonstrated that HIV infection is consistently associated with alterations in gut microbial communities. This dysbiosis is typically characterized by a loss of beneficial commensals and an expansion of pro-inflammatory taxa [13,14]. These taxonomic shifts are accompanied by profound functional metabolic changes, particularly involving short-chain fatty acids (SCFAs) and tryptophan catabolites [15]. Importantly, these dysbiotic patterns often persist even in individuals receiving effective ART with sustained virological suppression, suggesting that current therapies do not fully restore microbiome homeostasis. Beyond general inflammation, microbiome-driven systemic activation—specifically the chronic leakage of lipopolysaccharide (LPS) and other microbial products—is now recognized as a critical driver of specific non-AIDS-defining illnesses. For instance, sustained innate immune activation by translocated microbiota contributes directly to accelerated endothelial dysfunction and neuroinflammation. This significantly increases the risk of cardiovascular events and neurocognitive impairment in PLWH [16,17].

Recent advances in high-throughput sequencing and multi-omics technologies—including metagenomics, metatranscriptomics, metabolomics, and single-cell analyses—have significantly expanded our understanding of microbiome–host–virus interactions [18]. In parallel, experimental models such as germ-free mice, humanized mouse systems, and intestinal organoid platforms are providing valuable mechanistic insights into how specific microbial taxa and metabolites influence immune activation and viral persistence [19,20,21]. These approaches are helping move the field beyond descriptive associations toward identifying causal relationships.

From a therapeutic perspective, the gut microbiome represents a potentially modifiable target. Strategies aimed at restoring microbial balance—including probiotics, prebiotics, synbiotics, dietary interventions, and fecal microbiota transplantation (FMT)—are currently being explored as adjunctive approaches for improving mucosal immunity and reducing inflammation in PLWH. Some early studies suggest modest improvements in microbial diversity and specific immune parameters, such as normalized CD4^+^/CD8^+^ T-cell ratios, partial restoration of the Th17/Treg balance, and reductions in circulating pro-inflammatory cytokines like IL-6 and TNF-alpha. However, the available evidence remains preliminary and heterogeneous [22,23]. Emerging technologies, such as engineered bacterial therapeutics designed to deliver immunomodulatory molecules or influence viral latency, offer additional promising avenues but remain largely experimental at present [24].

Despite these advances, several important challenges remain. HIV-associated microbiome signatures vary considerably across geographic regions and demographic groups, reflecting differences in diet, sexual behavior, environmental exposures, and host genetics [13]. In addition, methodological variability—ranging from sample collection techniques to sequencing platforms and bioinformatic pipelines—continues to complicate comparisons across studies [25]. Perhaps most critically, the direction of causality remains unresolved: dysbiosis may actively contribute to immune dysfunction, but it may also arise as a consequence of HIV-induced mucosal damage. Clarifying this relationship will be essential for the development of effective microbiome-targeted therapies [26].

In this review, we synthesize current knowledge of microbiome–HIV interactions, with a particular focus on the mechanisms through which gut dysbiosis contributes to immune activation and viral persistence. We integrate findings from both human studies and experimental models, discuss emerging microbiome-directed therapeutic strategies, and highlight key gaps that remain in the field. Ultimately, a deeper understanding of host–microbiome–virus interactions may open new opportunities to complement ART and improve long-term outcomes for people living with HIV.

## 2. HIV and the Gut Immune System

### 2.1. Early CD4^+^ T-Cell Depletion in GALT

The gastrointestinal tract represents one of the earliest and most profoundly affected sites during acute HIV infection. Shortly after viral exposure, HIV preferentially targets CD4^+^ T cells within the gut-associated lymphoid tissue (GALT), leading to rapid and extensive depletion of these cells [10]. Among the most affected are Th17 and Th22 subsets, which play critical roles in maintaining epithelial barrier integrity and coordinating mucosal immune defenses [9]. Notably, the magnitude of CD4^+^ T-cell loss in the gut greatly exceeds that observed in peripheral blood [27,28]. This profound immunological disruption compromises epithelial regeneration, weakens mucosal protection against luminal microbes, and contributes to the development of systemic immune dysregulation.

### 2.2. Epithelial Barrier Dysfunction

The loss of protective mucosal T-cell populations is further compounded by both direct viral effects on intestinal epithelial cells and indirect damage mediated by inflammatory cytokines. Structural alterations in the gut barrier include disruption of tight junctions, epithelial cell apoptosis, and impaired mucosal repair mechanisms [29]. As a result, biomarkers indicative of epithelial injury remain elevated in people living with HIV (PLWH), even among those receiving suppressive antiretroviral therapy. These markers include intestinal fatty acid-binding protein (I-FABP), zonulin, and regenerating islet-derived protein 3 alpha (REG3α), all of which reflect ongoing epithelial damage and compromised barrier function [12,30].

### 2.3. Microbial Translocation

Damage to the intestinal barrier facilitates microbial translocation, a process in which microbial products escape from the gut lumen into systemic circulation. These products include bacterial and fungal components such as lipopolysaccharide (LPS), peptidoglycan, flagellin, and β-D-glucan [11]. Once in circulation, these pathogen-associated molecular patterns (PAMPs) activate innate immune receptors, including toll-like receptors (TLRs), thereby triggering sustained immune stimulation. Biomarkers of microbial translocation—most notably soluble CD14 (sCD14) and lipopolysaccharide-binding protein (LBP)—remain elevated even in individuals with effective ART-mediated viral suppression, indicating that microbial leakage persists despite virological control [15,31].

### 2.4. Chronic Immune Activation and Inflammation

Microbial translocation is a major driver of systemic immune activation, which is widely recognized as a defining feature of HIV pathogenesis and a strong predictor of disease progression and mortality [32]. Persistent immune activation promotes T-cell exhaustion, interferes with effective immune reconstitution, and supports the long-term maintenance of viral reservoirs [33]. Multiple inflammatory pathways remain chronically engaged, including type I interferon signaling, inflammasome activation, and increased expression of immune checkpoint molecules such as PD-1 and TIM-3 [34]. Importantly, chronic immune activation is closely associated with the development of non-AIDS comorbidities—including cardiovascular disease, liver fibrosis, and neurocognitive impairment—that disproportionately affect individuals receiving long-term ART [4,5].

### 2.5. ART and Incomplete Mucosal Immune Reconstitution

**Figure 1:**
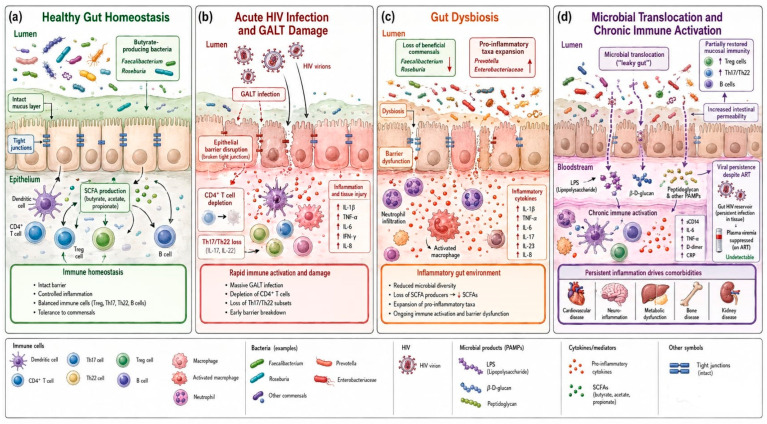
The HIV–gut–microbiome axis.

Although ART effectively suppresses plasma viremia and restores CD4^+^ T-cell counts in peripheral blood, it rarely results in complete restoration of mucosal immunity [35]. A substantial subset of individuals remains immunological non-responders (INRs), failing to achieve adequate CD4^+^ T-cell recovery despite sustained viral suppression [16]. Emerging evidence suggests that persistent gut dysbiosis and ongoing microbial translocation are associated with incomplete immune reconstitution, a characteristic of immunological non-responders (INRs) in these patients [36]. Consequently, incomplete mucosal immune reconstitution remains a major obstacle to achieving full immune normalization and may contribute to the continued morbidity observed in ART-treated populations (Figure 1).

Schematic overview of the mechanisms linking HIV infection to gut dysbiosis and systemic immune activation.

(a) In the healthy gut, commensal bacteria including butyrate-producing taxa maintain epithelial barrier integrity and mucosal immune homeostasis.
(b) Acute HIV infection results in rapid depletion of CD4^+^ T cells within the gut-associated lymphoid tissue (GALT), particularly Th17 and Th22 subsets, leading to epithelial barrier disruption and a prominent elevation (↑) of mucosal pro-inflammatory cytokines (IL-1 beta, TNF-alpha, IL-6, IFN-gamma, IL-8).
(c) Microbial dysbiosis emerges, characterized by depletion (↓) of beneficial commensals (e.g., *Faecalibacterium* and *Roseburia*) and enrichment (↑) of pro-inflammatory taxa such as *Prevotella* and *Enterobacteriaceae*.
(d) Increased gut permeability facilitates microbial translocation of bacterial products including lipopolysaccharide (LPS) and β-D-glucan into systemic circulation, driving chronic immune activation—marked by elevated (↑) plasma biomarkers (sCD14, IL-6, TNF-alpha, D-dimer, CRP)— and contributing to viral persistence and HIV-associated comorbidities despite antiretroviral therapy.

### 2.6. Influence of Biological Sex and Hormones on Mucosal Immunity

Biological sex is likely to influence HIV-associated dysbiosis and mucosal injury through direct effects of sex hormones on epithelial barrier integrity and immune regulation. Estrogen has been shown to strengthen epithelial barrier function by regulating tight junction proteins such as occludin, claudin-1, and zonula occludens-1 (ZO-1), whereas estrogen deficiency is associated with increased intestinal permeability [37,38]. Experimental studies further indicate that estradiol can preserve epithelial integrity under inflammatory conditions, supporting a protective role for estrogen signaling [37].

Sex hormones also influence mucosal immune responses. Estrogen signaling has been linked to maintenance of regulatory T-cell function and modulation of the Th17/Treg balance, both of which are essential for intestinal homeostasis [39]. In parallel, interactions between sex hormones and the gut microbiome may contribute to sex-dependent differences in microbial composition and inflammatory tone [40].

In the context of HIV infection—where epithelial damage and chronic immune activation are central features—these mechanisms may help explain why the severity of “leaky gut” and its consequences can vary between individuals. Hormonal status, environmental exposures, and baseline microbiome composition likely interact to shape disease progression. Crucially, this hypothesized link between biological sex and dysbiosis is strongly substantiated by real-world clinical and epidemiological cohort data, moving beyond animal models. Longitudinal data from large-scale human cohorts, most notably the Women’s Interagency HIV Study (WIHS), have consistently revealed that cisgender women living with HIV display distinct gut microbiome signatures and significantly higher baseline alpha-diversity compared to men under long-term, suppressive ART [13]. Furthermore, clinical plasma profiling has shown that women exhibit significantly lower circulating levels of key microbial translocation and innate activation markers, such as soluble CD14 (sCD14) and lipopolysaccharide-binding protein (LBP), even when adjusting for age and behavioral variables [13,31,41]. These real-world findings provide robust clinical evidence that sex-specific hormonal profiles—particularly estrogen-mediated tight junction stabilization—exert a tangible, protective effect against severe mucosal barrier disruption and systemic inflammation in people living with HIV.

## 3. Gut Microbiome Dysbiosis in HIV Infection

### 3.1. Changes in Microbial Composition

A growing number of studies have documented consistent alterations in the composition of the gut microbiota among people living with HIV (PLWH). In general, HIV-associated dysbiosis is characterized by a reduction in beneficial commensal bacteria alongside an expansion of microbial taxa linked to inflammatory processes.

Depleted taxa:

Among the most consistently reduced organisms are butyrate-producing bacteria, including *Faecalibacterium prausnitzii*, *Roseburia* species, and members of the *Ruminococcaceae* family [13,17,42]. These commensal microbes play an important role in maintaining epithelial barrier integrity and supporting mucosal immune balance.

Enriched taxa:

In contrast, several microbial groups associated with inflammation are commonly reported in higher abundance in HIV-infected individuals. These include *Prevotella* species, Enterococcus, and members of the *Enterobacteriaceae* family [13,15,43]. However, it is crucial to critically evaluate these findings in the context of confounding factors. Recent comparative studies have demonstrated that the enrichment of *Prevotella* and the depletion of *Bacteroides* or butyrate-producing taxa are strongly driven by sexual behavior—specifically men who have sex with men (MSM) status—diet, and frequent antibiotic exposure, rather than being exclusive signatures of HIV infection itself [13,44]. Conflicting studies have shown that HIV-negative MSM exhibit gut microbiome profiles strikingly similar to those of HIV-positive MSM, suggesting that lifestyle and behavioral factors can easily obscure true viral-induced dysbiosis [44].

Variability across populations:

Importantly, HIV-associated microbial signatures are not identical across all populations. Variations in diet, geographic location, and behavioral factors strongly influence microbiome composition, explaining the significant inconsistencies observed in the literature. These conflicting data highlight the fundamental complexity of defining a universal microbiome profile associated with HIV and underscore the necessity of matching control groups strictly by lifestyle and behavioral variables [13].

### 3.2. Functional and Metabolic Shifts

**Figure 2:**
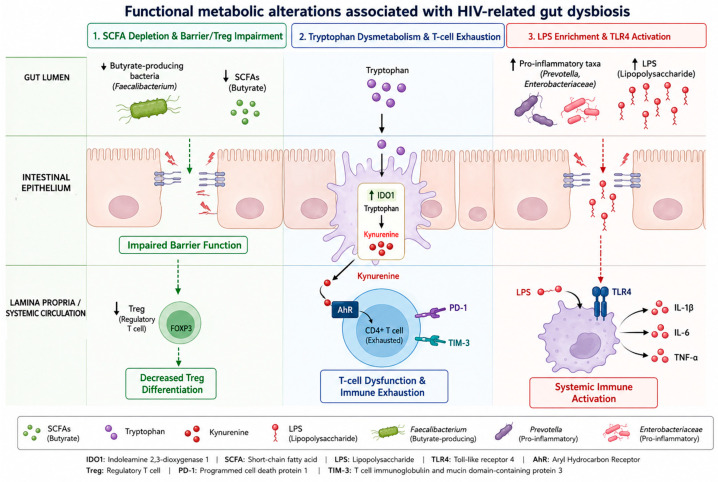
Functional Metabolic Alterations and Immune-Metabolic Crosstalk in HIV-Associated Gut Dysbiosis.

In addition to taxonomic changes, HIV-associated dysbiosis is accompanied by significant alterations in microbial metabolic activity. Insights from functional metagenomics and metabolomics analyses indicate that disruptions in microbial metabolism directly influence host immune responses. Key functional alterations include the marked depletion of SCFAs (particularly butyrate), dysregulated tryptophan catabolism via the indoleamine-2,3-dioxygenase (IDO1) pathway, and the enrichment of pro-inflammatory biosynthetic pathways such as LPS production (Figure 2). Crucially, these metabolic shifts trigger a bidirectional oxidative stress response that links host cellular dysfunction with microbial imbalance. On the host side, the chronic translocation of lipopolysaccharide (LPS) and the actions of viral accessory proteins (such as Tat and Nef) stimulate epithelial and immune cells to generate high levels of host-derived reactive oxygen species (ROS). This occurs primarily through the overactivation of nicotinamide adenine dinucleotide phosphate (NADPH) oxidase (NOX) pathways and localized mitochondrial decay [12,17]. This host-mediated oxidative stress leads to the accumulation of systemic pro-oxidant products, including lipid peroxidation markers like malondialdehyde (MDA). In turn, this altered, highly oxidized microenvironment directly impacts the microbiome: it selectively exerts oxygen toxicity against beneficial, strict anaerobic commensals (such as butyrate-producing *Faecalibacterium*), while driving the enrichment of specific bacterial pathways involved in oxidative stress defense, thereby permanently locking the ecosystem into a pro-inflammatory state. Rather than reiterating these complex processes here, the specific, synergistic mechanisms through which these metabolic shifts drive systemic immune activation and T-cell exhaustion are comprehensively discussed in Section 4 (Microbiome–Immunity–Virus Crosstalk).

Microbial metabolic pathways altered during HIV infection influence host immune responses. Loss of short-chain fatty acid (SCFA)–producing bacteria reduces the availability of metabolites such as butyrate that normally promote regulatory T-cell differentiation and maintain epithelial barrier integrity. Concurrently, dysregulated tryptophan metabolism through the indoleamine-2,3-dioxygenase (IDO) pathway increases kynurenine production, contributing to T-cell dysfunction and immune exhaustion. Enrichment of lipopolysaccharide (LPS) biosynthesis pathways further stimulates innate immune receptors such as Toll-like receptor 4 (TLR4), promoting systemic immune activation. These metabolic alterations collectively contribute to persistent inflammation in people living with HIV.

### 3.3. Geographic and Behavioral Influences

The composition of the gut microbiome in HIV infection varies considerably across populations. Studies have shown that microbial signatures differ depending on geographic location, dietary habits, and behavioral factors. For example, a global cohort study demonstrated that men who have sex with men (MSM) and individuals living in high-income versus low-income regions exhibit distinct microbial profiles [13]. These differences complicate efforts to define a universal pattern of HIV-associated dysbiosis.

Diet and lifestyle also play important roles in shaping microbial communities. Dietary patterns rich in fiber have been associated with partial restoration of beneficial microbial taxa and improved metabolic activity within the gut microbiome [45]. Such findings highlight the importance of considering environmental and behavioral factors when interpreting microbiome studies and designing targeted interventions.

### 3.4. Persistence Under ART

Although antiretroviral therapy effectively suppresses plasma viremia, it does not fully restore gut microbial homeostasis. Several studies have reported that individuals receiving long-term ART continue to exhibit microbial communities that differ substantially from those of HIV-negative individuals. These differences often include reduced alpha diversity and persistent alterations in microbial metabolic pathways [46].

Persistent dysbiosis has also been linked to incomplete immune reconstitution and elevated levels of systemic inflammation, suggesting that microbiome disturbances may contribute to the inability of ART alone to fully normalize host immune function [16,46]. Consequently, addressing microbiome imbalance may represent an important component of strategies aimed at improving long-term outcomes in people living with HIV. A comprehensive overview of the key taxonomic, metabolic, and clinical features characterizing HIV-associated dysbiosis is summarized in Table 1.

### 3.5. Beyond Bacteria: The Enteric Virome and Mycobiome in HIV

While most studies of HIV-associated dysbiosis have focused primarily on bacterial communities, it is increasingly recognized that the gut microbiome functions as a complex, multi-kingdom ecosystem in which viruses, bacteria, and fungi interact dynamically. These trans-kingdom interactions play a critical role in shaping microbial community structure, metabolic activity, and host immune responses, and are significantly altered during HIV infection [19].

Enteric virome and bacteriophage–bacteria interactions:

Recent studies have shown that HIV infection is associated with profound alterations in the enteric virome, characterized by an expansion of eukaryotic viruses and shifts in bacteriophage populations [19]. Bacteriophages dictate bacterial community structure through predator-prey dynamics and horizontal gene transfer. During HIV infection, an altered phage-to-bacteria ratio can trigger the lytic destruction of beneficial commensal bacteria, inadvertently facilitating the expansion of pro-inflammatory bacterial pathobionts. This phage-mediated structural collapse of the bacterial microbiome fundamentally accelerates mucosal inflammation and ecosystem instability.

Mycobiome, *Candida*-related immune activation, and beta-D-glucan:

Fungal dysbiosis is another critical driver of mucosal immune dysfunction. HIV infection is frequently associated with an overgrowth of Candida species in the gastrointestinal tract. Consequent to epithelial barrier breakdown, fungal structural components—most notably beta-D-glucan (BDG)—translocate into the systemic circulation [12]. Circulating BDG serves as a highly robust biomarker for gut damage and systemic fungal translocation. Upon translocation, BDG strongly binds to pattern recognition receptors such as Dectin-1 and various Toll-like receptors on innate immune cells (e.g., macrophages and dendritic cells). This interaction triggers a potent inflammatory cascade, resulting in the massive release of pro-inflammatory cytokines such as IL-1 beta, IL-6, and TNF-alpha [12,19]. Elevated levels of circulating fungal products have been explicitly linked to clinical markers of monocyte activation and non-AIDS comorbidities, confirming that fungal dysbiosis plays an active role in driving persistent host inflammation [12,49]. This clinical relevance is further underscored by recent clinical cohort data from severely immunosuppressed individuals (CD4^+^ T-cell counts < 200 cells/uL), which demonstrate a distinct shift toward *Candida*-dominated mycobiotypes characterized by the marked enrichment of *Candida albicans*, *Candida dubliniensis*, and *Nakaseomyces glabratus*, directly linking advanced immune depletion to systemic fungal translocation risks [50].

Influence on HIV persistence:

Crucially, these non-bacterial components actively contribute to the maintenance of the latent HIV reservoir. The chronic innate immune activation and elevated cytokine levels driven by circulating BDG and virome disturbances create a highly inflammatory tissue microenvironment. This milieu promotes the homeostatic proliferation and survival of memory CD4^+^ T cells that harbor latent proviruses. This directly links multi-kingdom dysbiosis to viral persistence despite effective ART [12].

## 4. Microbiome–Immunity–Virus Crosstalk

### 4.1. Dysbiosis and Chronic Immune Activation

HIV-associated dysbiosis plays a central role in sustaining chronic immune activation, not only through compositional changes but also via alterations in microbial metabolites and host signaling pathways. A reduction in butyrate-producing bacteria leads to decreased availability of short-chain fatty acids (SCFAs), which are essential for regulatory T cell (Treg) differentiation and for limiting excessive inflammatory responses [15,51].

At the same time, expansion of pro-inflammatory taxa—such as *Prevotella* species and members of the *Enterobacteriaceae* family—results in increased production of microbial components including lipopolysaccharide (LPS). These molecules can activate innate immune receptors such as toll-like receptor 4 (TLR4), thereby promoting sustained systemic inflammation [11,15,17].

Importantly, these processes are closely interconnected. Reduced anti-inflammatory signaling and increased exposure to microbial products act together to maintain a state of persistent immune activation, even in individuals receiving effective antiretroviral therapy (ART). This suggests that dysbiosis is not simply a consequence of HIV infection but an active driver of immune dysregulation.

### 4.2. Tryptophan Catabolism, SCFA Crosstalk, and T-Cell Dysfunction

Alterations in microbial and host metabolism during HIV infection also influence immune regulation through the tryptophan pathway. Increased activity of the enzyme indoleamine 2,3-dioxygenase (IDO1) promotes the degradation of tryptophan into kynurenine metabolites [48]. Crucially, IDO1 hyperactivation and SCFA depletion are not isolated events but form a synergistic regulatory network. In a healthy gut, SCFAs such as butyrate suppress IDO1 expression in intestinal epithelial cells and dendritic cells through histone deacetylase (HDAC) inhibition and STAT regulatory pathways. The loss of SCFA-producing bacteria in HIV infection removes this inhibitory control, directly driving IDO1 overactivation.

The resulting accumulation of kynurenine further exacerbates dysbiosis. Kynurenine and its downstream pro-oxidant metabolites can induce a hostile microenvironment that inhibits the growth of beneficial, oxygen-sensitive butyrate producers, creating a vicious feed-forward loop. At the molecular level, translocated kynurenine directly impairs mucosal and systemic immunity by serving as a high-affinity endogenous ligand for the Aryl Hydrocarbon Receptor (AhR), a cytosolic transcription factor heavily expressed in T cells [47,48]. Upon entering naive CD4^+^ T cells, kynurenine binds to AhR, triggering its translocation into the nucleus. This transcriptional activation promotes STAT5 phosphorylation and directly upregulates FoxP3 expression, thereby forcibly driving the differentiation of naive T cells into immunosuppressive regulatory T cells (Tregs). Crucially, this occurs at the expense of protective, mucosal-stabilizing Th17 and Th22 cell subsets [47]. Furthermore, chronic AhR hyperactivation by kynurenine leads to the progressive upregulation of inhibitory immune checkpoints, including programmed cell death protein 1 (PD-1), T-cell immunoglobulin and mucin-domain containing-3 (TIM-3), and lymphocyte-activation gene 3 (LAG-3) on effector T cells. As a result, this synergistic metabolic disruption—where SCFA loss fuels IDO1 activity, and kynurenine accumulation drives intracellular AhR signaling—profoundly drives persistent immune dysfunction, T-cell exhaustion, and poor immune recovery in PLWH [48].

### 4.3. Immune Checkpoints and T-Cell Exhaustion

Chronic exposure to microbial antigens and inflammatory mediators further promotes immune dysregulation through the upregulation of inhibitory receptors on T cells. Molecules such as PD-1, TIM-3, and LAG-3 are commonly expressed on chronically stimulated T cells and are widely recognized as hallmarks of T-cell exhaustion [34].

This exhausted phenotype limits the ability of T cells to mount effective antiviral responses and has been strongly linked to the persistence of latent viral reservoirs [52].

From a broader perspective, these findings suggest that dysbiosis-driven immune activation may not only sustain inflammation but also reinforce immune checkpoint pathways that favor viral persistence. This highlights a potential intersection between microbiome dynamics and emerging immunotherapeutic strategies targeting checkpoint pathways.

### 4.4. Influence on HIV Reservoirs

Microbial translocation and gut dysbiosis may contribute to the maintenance of HIV reservoirs through several interconnected mechanisms. Pro-inflammatory cytokines such as IL-6 and TNF-α can promote homeostatic proliferation of infected CD4^+^ T cells, thereby expanding the pool of cells harboring latent virus [2]. In parallel, persistent immune activation sustains inflammatory tissue environments—particularly within lymphoid and gut compartments—that support long-term viral persistence [46]. Together, these processes create conditions that favor the stability and expansion of the latent HIV reservoir.

Beyond immune activation, microbial metabolites play an important role in the epigenetic regulation of HIV latency. Short-chain fatty acids (SCFAs), particularly butyrate, function as endogenous histone deacetylase (HDAC) inhibitors. HDAC enzymes normally promote chromatin condensation and transcriptional repression; thus, their inhibition leads to increased histone acetylation and a more transcriptionally permissive chromatin state.

In this context, butyrate-mediated HDAC inhibition can facilitate access of transcriptional machinery to the HIV long terminal repeat (LTR), promoting transcriptional activation of latent proviruses. This mechanism closely resembles the “shock” phase of the “shock and kill” strategy, in which latency-reversing agents are used to induce viral expression and expose infected cells to immune clearance [51,53].

However, HIV-associated dysbiosis is typically characterized by a depletion of butyrate-producing bacteria, which may reduce endogenous HDAC inhibition and favor the persistence of transcriptionally silent reservoirs. At the same time, localized SCFA production within specific gut niches may still influence latency dynamics, suggesting that microbiome effects on HIV persistence are spatially and functionally complex.

Taken together, these findings point to the microbiome as a potential modulator of HIV latency. Therapeutic strategies aimed at restoring SCFA-producing bacteria or delivering microbiome-derived metabolites may enhance the effectiveness of latency-reversing approaches and contribute to reservoir reduction.

### 4.5. ART–Microbiome Interactions

The relationship between the gut microbiome and antiretroviral therapy (ART) is increasingly recognized as bidirectional, involving complex interactions that influence both drug efficacy and host physiology. While ART can reshape microbial composition, emerging evidence suggests that the microbiome itself can modulate drug metabolism, toxicity, and long-term treatment outcomes [14,54].

On one hand, antiretroviral drugs—particularly protease inhibitor–based regimens—have been associated with reduced microbial diversity and enrichment of pro-inflammatory taxa. These changes may contribute to metabolic complications such as insulin resistance and dyslipidemia observed during long-term therapy [14,17]. In contrast, integrase inhibitors appear to have a more limited impact on microbiome composition, although available data remain relatively sparse [14].

On the other hand, microbial communities can influence the pharmacokinetics of antiretroviral drugs. Certain bacterial species possess enzymatic pathways capable of metabolizing nucleoside reverse transcriptase inhibitors (NRTIs), potentially altering drug bioavailability and therapeutic efficacy [54]. In addition, microbial metabolites—such as SCFAs and bile acid derivatives—can modulate host metabolic pathways and drug responses [45,55].

Importantly, microbiome composition may also contribute to ART-associated toxicities. Dysbiosis-driven inflammation has been implicated in the development of cardiovascular complications, a major non-AIDS comorbidity in PLWH [4,5]. Microbial products such as LPS may exacerbate endothelial dysfunction and promote atherogenesis through activation of innate immune pathways [4,5,11].

Similarly, alterations in the gut microbiome may plausibly influence bone metabolism through inflammatory and metabolic pathways [51,55].

Together, these findings highlight the emerging field of pharmacomicrobiomics, in which host–microbiome interactions shape drug response, toxicity, and long-term clinical outcomes. A better understanding of these interactions may enable more personalized and effective HIV treatment strategies.

### 4.6. The Gut–Brain Axis and Neuroinflammation

Microbiome-driven inflammation extends beyond the gastrointestinal tract, profoundly impacting the central nervous system (CNS) through the gut–brain axis. This bidirectional communication network is particularly relevant to the pathogenesis of HIV-associated neurocognitive disorders (HAND) [5]. The mechanisms linking gut dysbiosis to neurocognitive impairment systematically involve microbial metabolites, immune cell migration, and vagus nerve signaling [56].

First, the depletion of SCFAs deprives the CNS of critical neuroprotective signals. In a healthy state, gut-derived SCFAs can cross the blood–brain barrier (BBB) to maintain microglia in a resting state and promote neurogenesis; their absence in HIV infection facilitates microglial hyperactivation and neurotoxicity [15,51]. Second, gut barrier dysfunction and systemic LPS translocation trigger the activation and expansion of pro-inflammatory monocytes (such as the CD14^+^CD16^+^ subsets) in the intestinal mucosa [11,12]. These gut-primed immune cells traffic through systemic circulation and cross the BBB, seeding the CNS with inflammatory cytokines and latent HIV [57]. Finally, the enteric nervous system detects dysbiosis-induced mucosal inflammation, transmitting stress signals to the brain via vagus nerve afferents, which further amplifies neuroinflammation [56].

Consequently, microbiota-targeted interventions—such as specialized diets or engineered biotherapeutics—restore SCFA production, dampen vagus nerve inflammatory signaling, and reduce the migration of activated monocytes. These strategies hold significant promise for modulating the gut–brain axis and mitigating neurocognitive decline in PLWH [22,24].

## 5. Experimental Models and Tools

### 5.1. Germ-Free and Gnotobiotic Mouse Models

Germ-free mice, which are raised in sterile environments and lack endogenous microbiota, provide a powerful system for studying the role of microbial communities in disease. By colonizing these animals with fecal microbiota derived from people living with HIV (PLWH), researchers can examine how HIV-associated microbial communities influence immune activation, metabolism, and host physiology [20]. Gnotobiotic models—where defined microbial consortia are introduced—allow even more precise investigation of specific taxa, such as butyrate-producing bacteria or Prevotella species, and their roles in shaping immune responses [58].

However, these models possess significant translational limitations. Germ-free mice suffer from inherently defective intestinal architecture and underdeveloped immune systems due to the absence of microbial stimulation during early development. They exhibit hypoplastic gut-associated lymphoid tissues (GALT), reduced secretory IgA production, and an altered balance of Th17 and Treg cells. Furthermore, their barrier integrity is structurally compromised, characterized by a significantly thinner mucus layer and atypical tight junction protein expression compared to conventionally raised mice [26]. Consequently, while germ-free mice are excellent for isolating the effects of specific microbes, their basal physiological state does not accurately simulate the mature, microbially educated human intestinal microenvironment.

### 5.2. Humanized Mouse Models

Humanized mouse models, in which mice are engrafted with human immune cells and tissues, enable the study of HIV infection within a human-like immune environment [59,60]. When combined with experimental manipulation of the gut microbiome, these systems provide insights into dysbiosis and mucosal immune responses [61].

Despite their utility, humanized mice present inherent defects in simulating the human gastrointestinal tract. A major limitation is the species mismatch: engrafted human immune cells must interact with a murine intestinal epithelium and murine microbiota. This lack of species-specific human immune-epithelial crosstalk severely restricts the proper modeling of barrier integrity and microbial translocation. Additionally, humanized mice often suffer from incomplete immune cell reconstitution. They typically lack a robust and functional repertoire of human mucosal innate immune cells, such as neutrophils, dendritic cells, and specialized tissue-resident macrophages, which are critical first responders to gut dysbiosis [59]. Furthermore, incomplete human leukocyte antigen (HLA) matching between the human hematopoietic stem cells and the murine thymus can result in defective T-cell education and abnormal immune responses. Therefore, findings from humanized mice must be interpreted with caution when translating microbiome-immune dynamics to human HIV pathogenesis.

### 5.3. Non-Human Primate Models

Non-human primate models, particularly simian immunodeficiency virus (SIV) infection in macaques, are widely regarded as the gold standard for studying HIV pathogenesis. SIV infection in macaques closely mirrors many features of human HIV infection, including rapid depletion of gut CD4^+^ T cells, microbial translocation, and microbiome disruption [62]. These models have played a critical role in testing interventions such as probiotics, dietary modulation, and fecal microbiota transplantation (FMT) in the context of HIV/SIV infection [63].

### 5.4. Intestinal Organoids and Ex Vivo Systems

Three-dimensional intestinal organoids derived from stem cells provide an advanced platform for modeling the human intestinal epithelium. These organoids reproduce key aspects of the gut architecture and function and can be co-cultured with immune cells and microbes to study host–microbe interactions under controlled conditions [21]. In parallel, ex vivo lamina propria mononuclear cell (LPMC) cultures obtained from HIV-infected individuals allow direct examination of immune responses within human intestinal tissues [57]. Together, these experimental systems enable detailed investigation of how microbial metabolites and HIV infection affect epithelial integrity and immune signaling.

### 5.5. Multi-Omics Platforms

The growing availability of multi-omics datasets has introduced significant analytical complexity, creating an important role for artificial intelligence (AI) and machine learning in microbiome research. These approaches enable the identification of microbial patterns associated with clinical outcomes that may not be detectable using traditional statistical methods [36,64]. In addition, network-based computational approaches can help uncover interactions between microbial taxa, metabolites, and host immune pathways.

## 6. Therapeutic Potential of Targeting the Microbiome

When evaluating microbiome-targeted interventions, it is critical to distinguish their varying degrees of translational maturity and evidence quality. Currently, strategies such as probiotics and prebiotics are supported by small randomized controlled trials (RCTs) in humans, whereas fecal microbiota transplantation (FMT) remains in the early clinical pilot stage for HIV. Conversely, next-generation approaches like engineered microbial therapeutics are strictly confined to preclinical animal models. Therefore, the clinical applicability of these interventions spans a wide spectrum from experimental proof-of-concept to early clinical testing.

### 6.1. Probiotics and Prebiotics

**Figure 3:**
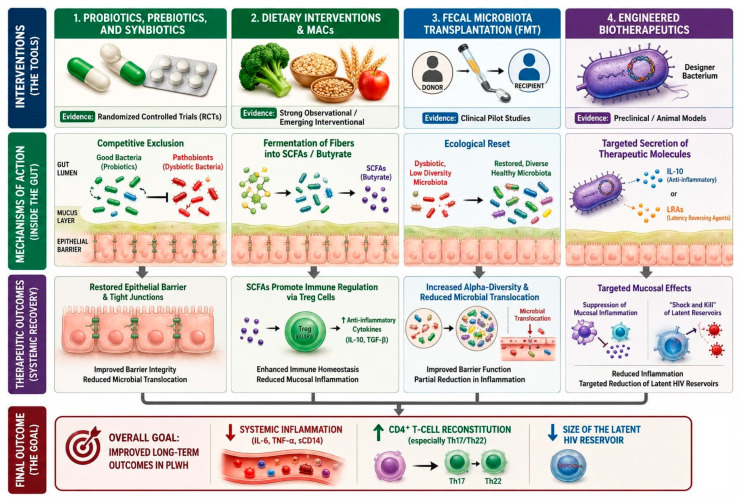
Microbiome-targeted therapeutic strategies in HIV infection: Mechanisms of action and translational evidence.

Probiotics—live microorganisms that confer health benefits to the host—have been explored in several small clinical studies involving people living with HIV (PLWH). Supplementation with strains such as *Lactobacillus* and *Bifidobacterium* has been associated with partial restoration of microbial diversity, reductions in markers of microbial translocation, and, in some cases, modest improvements in CD4^+^ T-cell recovery [22,23]. These microbiome-targeted strategies and their proposed mechanisms are summarized in Figure 3.

That said, the clinical effects of probiotics are not entirely consistent and the literature is plagued by significant methodological flaws. Many early clinical trials suffer from a lack of rigorous, randomized placebo-controlled designs, making it difficult to distinguish true therapeutic efficacy from placebo effects [8]. Furthermore, extreme outcome heterogeneity—with studies utilizing wildly different immunological markers, probiotic strains, and dosing regimens—precludes standardized conclusions. This variability likely reflects differences in study design, treatment duration, and individual host factors, including baseline microbiome composition and ART status [22,65].

Prebiotics, such as inulin and fructooligosaccharides, aim to promote the growth of beneficial bacteria by providing fermentable substrates. When used alongside antiretroviral therapy (ART), they have been linked to modest increases in short-chain fatty acid (SCFA) production and some improvement in markers of gut barrier integrity [66]. However, these effects are generally modest, and their clinical significance remains uncertain due to the aforementioned methodological shortcomings [45].

### 6.2. Synbiotics and Dietary Interventions

Synbiotics combine probiotics with prebiotics in an effort to enhance their overall effect. Theoretically, the rationale for synbiotics relies on achieving a true statistical “synergistic effect” (i.e., 1 + 1 > 2), where the combined intervention yields benefits vastly superior to the sum of its individual components. However, it is crucial to strictly distinguish this from a mere “additive effect” (i.e., 1 + 1 = 2). Although some early studies suggest that synbiotics may improve microbial richness and reduce inflammatory markers more effectively than probiotics alone [67], robust clinical evidence demonstrating true synergistic effects in PLWH remains very limited. Many observed benefits in current trials likely represent additive effects rather than true biological synergy. Even so, it is still unclear whether these microbiological changes translate into meaningful clinical benefits over the long term.

Diet is another important—and often overlooked—modulator of the gut microbiome. Diets rich in fiber may promote the abundance of SCFA-producing bacteria and beneficial metabolic activity within the gut microbiome [45]. Compared to supplementation strategies, dietary interventions may offer a more sustainable and physiologically relevant approach. However, their effectiveness is influenced by multiple factors, including adherence, baseline dietary habits, and environmental context.

### 6.3. Fecal Microbiota Transplantation (FMT)

Fecal microbiota transplantation (FMT) has gained attention as a strategy to restore microbial diversity in HIV infection. In pilot studies involving ART-treated individuals, FMT has been associated with increased bacterial richness and partial recovery of butyrate-producing taxa [68]. However, its impact on systemic immune activation has been inconsistent [69,70]. This suggests that simply increasing microbial diversity may not be sufficient to reverse the underlying immune dysfunction associated with HIV.

These findings also raise questions about how well transplanted microbiota integrate into the host environment. Factors such as donor–recipient compatibility, microbiome stability, and host immune status are likely to influence treatment outcomes. In addition, practical and safety concerns—particularly the risk of pathogen transmission and variability in protocols—remain important considerations in immunocompromised populations [70].

### 6.4. Engineered Microbes and Next-Generation Biotherapeutics

Advances in synthetic biology are opening new possibilities for microbiome-based therapies. Engineered microbial strains can be designed to deliver therapeutic molecules directly within the gut, modulate immune pathways, or produce anti-inflammatory compounds [71].

For instance, engineered bacterial strains such as *Lactococcus lactis* engineered to secrete IL-10 have demonstrated anti-inflammatory effects in experimental models [72]. These approaches offer a level of precision that is difficult to achieve with traditional probiotics. However, their clinical application remains at an early stage, and several challenges—including safety, regulatory approval, and long-term stability—need to be addressed before they can be widely implemented in HIV treatment.

### 6.5. Clinical Evidence and Limitations

Despite growing interest in microbiome-targeted interventions, the available clinical evidence in HIV remains notably weak and largely preliminary. The field suffers from pervasive methodological flaws; many studies are underpowered, lack rigorous placebo controls, and utilize highly heterogeneous outcome measures (ranging from specific cytokine levels to general diversity indices). This makes meta-analytical conclusions nearly impossible [8].

While approaches such as probiotics and FMT can sometimes successfully improve luminal microbial diversity, a glaring paradox remains: this taxonomic restoration rarely translates into consistent reductions in systemic inflammation or meaningful CD4^+^ T-cell recovery. This profound disconnect likely stems from a combination of treatment limitations and incomplete mechanistic understanding. First, therapeutic microbes often exhibit only transient engraftment; they fail to permanently colonize the hostile, inflammatory gut environment of PLWH. Consequently, any modest immune benefit vanishes once supplementation ceases. Second, merely increasing taxonomic diversity does not guarantee the restoration of critical metabolic functions, such as sufficient SCFA production required to heal the epithelial barrier. Finally, and perhaps most importantly, chronic HIV infection induces irreversible structural damage to the gut architecture. Prolonged viral replication leads to extensive collagen deposition and fibrosis within the GALT, permanently scarring the lymphatic tissue [29,73]. Consequently, even if luminal dysbiosis is perfectly corrected through FMT or probiotics, the underlying fibrotic scarring presents a physical barrier to CD4^+^ T-cell reconstitution and mucosal healing. This suggests that microbiome modulation alone is insufficient and must be combined with anti-fibrotic or potent anti-inflammatory therapies to achieve clinically relevant immune recovery.

Additional challenges include significant inter-individual variability in microbiome composition, an incomplete understanding of host–microbiome interactions, and evolving regulatory frameworks. Addressing these issues will require larger, well-designed clinical trials with standardized methodologies. Such efforts will be essential to determine whether microbiome-targeted therapies can move beyond supportive roles and become an integral part of HIV treatment and cure strategies. A summary of these emerging microbiome-targeted interventions, including their proposed mechanisms, key clinical findings, and current levels of evidence, is provided in Table 2.

Conceptual overview of emerging interventions aimed at modulating the gut microbiome to improve immune recovery and reduce inflammation in people living with HIV. Approaches include probiotics, prebiotics, and synbiotics to enhance beneficial microbial populations; dietary interventions to increase microbiota-accessible carbohydrates and short-chain fatty acid production; fecal microbiota transplantation (FMT) to restore microbial diversity; and engineered microbial therapeutics designed to deliver immunomodulatory molecules. These strategies may complement antiretroviral therapy and potentially enhance HIV cure approaches by reducing immune activation and improving mucosal immune function.

## 7. Challenges and Knowledge Gaps

### 7.1. Inter-Individual and Geographic Variability

HIV-associated dysbiosis does not manifest uniformly across different populations. Numerous studies have demonstrated substantial variability in microbiome composition depending on factors such as geographic location, dietary patterns, and sexual behavior [13]. For example, men who have sex with men (MSM) often exhibit microbial profiles that differ from those observed in heterosexual populations, regardless of HIV status [44]. Likewise, differences in diet between high-income and low-income regions can result in markedly distinct gut microbial communities.

These observations highlight an important challenge: the absence of a consistent “HIV-associated microbiome signature.” Rather than reflecting a single pattern, dysbiosis in HIV appears to be shaped by a combination of viral, environmental, and host-specific factors. This variability complicates efforts to develop standardized microbiome-based interventions and suggests that more personalized approaches may be required.

Overall, these findings emphasize the need to account for demographic and biological variability in microbiome research. Furthermore, as discussed in Section 2.6, biological sex and hormonal status—particularly estrogen’s regulatory effects on tight junctions and immune modulation—significantly impact epithelial barrier integrity and contribute to this inter-individual variability. Future studies should incorporate sex-specific and population-specific analyses to better understand heterogeneity in disease outcomes and treatment responses.

### 7.2. Causality Versus Correlation

A major unresolved question in the field is whether microbiome alterations actively contribute to HIV persistence and immune activation, or whether they arise primarily as a consequence of HIV-associated immune dysfunction and antiretroviral therapy. A significant flaw in the current literature is that the vast majority of human microbiome studies in HIV rely on cross-sectional designs [36]. Cross-sectional studies only capture a single snapshot in time, failing to account for the dynamic, longitudinal shifts in the microbiome that occur before, during, and after seroconversion or ART initiation. This inherent design limitation makes it virtually impossible to definitively separate cause from effect or to filter out the noise of transient environmental exposures.

While experimental models suggest that microbial communities can influence mucosal immunity, translating these findings to human populations remains challenging. The relationship is likely bidirectional: immune dysfunction may drive dysbiosis, while dysbiosis may further amplify immune activation.

Addressing this question will require well-designed longitudinal studies and interventional trials that can distinguish cause from consequence. Clarifying this relationship is critical for determining whether microbiome-targeted therapies can meaningfully alter disease progression.

### 7.3. Methodological Heterogeneity and Sampling Limitations

Another major challenge driving inconsistencies in the literature is extreme methodological variability, particularly regarding sampling strategies and sequencing technologies [25].

First, the choice between stool-based and mucosal sampling severely impacts study outcomes. The vast majority of studies rely on fecal samples due to their non-invasive nature. However, stool samples predominantly reflect the luminal microbiome and transient dietary residues. They do not accurately represent the mucosa-adherent microbial communities, which are biologically far more relevant because they directly interact with the gut epithelium, immune cells, and viral reservoirs [36]. While mucosal biopsies provide a more accurate picture of the host-microbe interface, they are invasive, difficult to obtain longitudinally, and susceptible to varied bowel preparation protocols, which themselves alter the microbiome.

Second, discrepancies arise from the sequencing platforms utilized. Many earlier studies relied on 16S rRNA gene amplicon sequencing, which provides only genus-level taxonomic resolution, suffers from PCR amplification biases, and relies on predictive algorithms (e.g., PICRUSt) to infer functional pathways. In contrast, shotgun metagenomics provides species- and even strain-level resolution while allowing for direct functional and metabolic profiling [25]. However, shotgun metagenomics is more expensive, requires intensive bioinformatic resources, and is highly sensitive to host DNA contamination, especially in mucosal biopsy samples.

Moving forward, greater standardization—such as utilizing multi-compartment sampling (matched stool and biopsies) and transitioning universally to shotgun metagenomic and metabolomic platforms—will be essential to resolve current literature conflicts and identify reliable microbial signatures.

### 7.4. Limited Clinical Evidence for Interventions

Although microbiome-targeted interventions such as probiotics, prebiotics, and fecal microbiota transplantation (FMT) show promise, robust clinical evidence remains limited. Many studies involve small cohorts, short follow-up periods, and heterogeneous outcome measures, making it difficult to draw definitive conclusions regarding clinical efficacy [8].

Moreover, while some interventions improve microbial diversity, their impact on key clinical outcomes—such as immune activation or viral reservoirs—remains inconsistent. This suggests that microbiome modulation alone may not be sufficient and may need to be combined with other therapeutic strategies.

In addition, long-term safety data—particularly for interventions such as FMT in immunocompromised populations—remain limited, underscoring the need for carefully designed clinical trials.

### 7.5. Ethical and Regulatory Considerations

The use of microbiome-based therapeutics introduces a range of ethical and regulatory challenges. Interventions involving live microorganisms, including FMT and engineered bacterial therapies, require rigorous donor screening and monitoring to minimize the risk of pathogen transmission [70].

Furthermore, the long-term ecological consequences of altering the gut microbiome are not yet fully understood. Regulatory agencies are still developing frameworks for evaluating and approving microbiome-based therapies, which may slow their integration into clinical practice.

Addressing these challenges will be essential to ensure both the safety and sustainability of microbiome-based interventions in HIV.

### 7.6. Integration with HIV Cure Research

An additional challenge lies in integrating microbiome modulation with emerging HIV cure strategies. Current approaches—including latency-reversing agents, immune checkpoint inhibitors, and gene-editing technologies—primarily target viral persistence and immune dysfunction. However, the potential role of the microbiome in modulating these interventions remains insufficiently explored.

Preclinical studies suggest that microbial metabolites can influence viral latency and immune exhaustion [34,52,75], pointing to a possible role of the microbiome in shaping treatment responses. However, clinical data are still limited, and the extent to which these findings translate to human populations remains unclear.

Developing effective combination strategies will require multidisciplinary research that integrates microbiology, immunology, and virology. Such approaches may ultimately provide more comprehensive and durable solutions for HIV treatment and cure.

## 8. Future Directions and Conclusions

### 8.1. Precision Microbiome Medicine

Future research should increasingly focus on precision approaches that account for inter-individual variability in microbiome composition. Integrating microbiome sequencing with host genetic information, dietary data, and immune profiling may enable the development of more personalized therapeutic strategies tailored to individual patients [76]. In this context, large-scale, multi-ethnic cohort studies will be essential for distinguishing universal microbiome features from population-specific patterns associated with HIV infection.

### 8.2. Multi-Omics and Systems Biology

The integration of high-throughput technologies—including metagenomics, metabolomics, transcriptomics, and proteomics—offers a powerful systems-level framework for studying host–microbe–virus interactions. Applying these approaches in longitudinal studies of both ART-treated and untreated individuals may help clarify causal relationships between microbiome alterations and HIV pathogenesis [76].

In addition, emerging tools such as single-cell sequencing and spatial transcriptomics are expected to provide more detailed insights into how microbial metabolites influence immune cell function and viral reservoirs within specific tissue environments. These advances may help bridge the gap between descriptive microbiome data and mechanistic understanding.

### 8.3. Novel Microbiome-Based Therapeutics

Beyond conventional approaches such as probiotics and fecal microbiota transplantation, the next generation of microbiome-based therapies is likely to involve engineered microbial systems. Advances in synthetic biology now allow the design of bacterial strains capable of producing anti-inflammatory cytokines, modulating immune pathways, or delivering latency-reversing agents directly within the gut environment [77].

While these approaches remain largely experimental, they offer a level of specificity and controllability that may overcome some of the limitations of traditional microbiome interventions. Combining such strategies with existing ART regimens or immunotherapeutic approaches could further enhance their potential in HIV treatment and cure research.

### 8.4. Integration with HIV Cure Strategies

Future research should systematically explore the role of microbiome modulation as an adjunct to HIV cure strategies. For example, restoring SCFA-producing microbial communities may help reduce immune activation and improve the effectiveness of latency-reversing agents. Similarly, targeting dysregulated tryptophan metabolism could alleviate T-cell exhaustion and enhance responses to immune checkpoint blockade therapies [48,51,78,79].

However, these concepts remain largely theoretical, and their clinical relevance has yet to be fully established. Advancing this field will require innovative experimental models and carefully designed clinical trials that directly assess the impact of microbiome modulation on viral reservoirs and immune function.

### 8.5. Ethical, Regulatory, and Equity Considerations

As microbiome-based therapies continue to develop, ethical and regulatory considerations must remain central to their implementation. Ensuring rigorous donor screening procedures, long-term safety monitoring, and clearly defined regulatory frameworks will be critical for safe clinical translation.

In addition, given that HIV disproportionately affects populations in low- and middle-income countries, future interventions must be not only effective but also accessible, affordable, and adaptable to diverse healthcare settings [80]. Addressing these issues will be essential to ensure equitable translation of microbiome-based innovations into clinical practice.

## 9. Conclusions

The gut microbiome is increasingly recognized as a key factor in HIV pathogenesis, influencing immune activation, viral persistence, and treatment outcomes. HIV-associated dysbiosis—characterized by the loss of beneficial commensal bacteria, expansion of pro-inflammatory taxa, and disruption of microbial metabolic pathways—persists even in individuals receiving effective antiretroviral therapy and contributes to incomplete immune reconstitution, a characteristic of immunological non-responders (INRs).

Recent advances in experimental models and multi-omics technologies have begun to clarify the complex interactions between the microbiome, host immunity, and viral persistence. At the same time, early clinical studies suggest that microbiome-targeted interventions may offer promising adjunctive strategies for improving immune function and reducing chronic inflammation in people living with HIV.

Nevertheless, several important challenges remain, including substantial variability in microbiome composition, methodological heterogeneity across studies, and limited clinical evidence for microbiome-based therapies. Addressing these challenges will require standardized methodologies, large-scale longitudinal studies, and close collaboration across disciplines.

Looking ahead, microbiome modulation represents an emerging therapeutic frontier that may complement existing HIV treatments, enhance immune recovery, and potentially contribute to strategies aimed at achieving durable HIV remission or eradication. Continued research into host–virus–microbiome interactions will be essential for translating these insights into meaningful clinical advances.

## Figures and Tables

**Table 1:** Characteristics of HIV-Associated Gut Microbiome Dysbiosis.

Feature	HIV-Negative/Healthy State	HIV-Associated Alterations	Functional Consequences	Key References
Taxonomic Composition				
Diversity	High alpha- and beta-diversity	Reduced alpha-diversity, distinct beta-diversity	Reduced ecological stability and functional redundancy	[13,17,46]
Beneficial Commensals	Abundant *Faecalibacterium*, *Roseburia*, Ruminococcaceae	Significant depletion	Loss of SCFA production, impaired barrier function	[42,43]
Pro-inflammatory Taxa	Balanced representation	Enriched *Prevotella*, *Enterobacteriaceae*, *Enterococcus*	Increased LPS production, mucosal inflammation	[15,43]
Metabolic Functions				
SCFA Production	Robust butyrate, acetate, propionate production	Markedly impaired SCFA biosynthesis	Reduced Treg differentiation, increased gut permeability	[15,17,46]
Tryptophan Metabolism	Balanced serotonin pathway	Shifted toward kynurenine pathway (IDO-mediated)	T-cell suppression, increased immune exhaustion	[47,48]
Biosynthetic Pathways	Diverse metabolic capabilities	Enriched LPS biosynthesis pathways	Chronic immune activation via TLR4 signaling	[15,17]
Clinical Correlates				
Microbial Translocation	Minimal	Elevated LPS, sCD14, LBP	Systemic immune activation, inflammation	[11,15,31]
Immune Recovery	Normal CD4^+^ reconstitution	Incomplete immune reconstitution (INRs)	Association with persistent dysbiosis	[16,36,46]
Therapeutic Response	N/A	Incomplete normalization with ART	Persistent dysbiosis despite virologic suppression	[16,17,46]

Note: Alpha-diversity measures the microbial richness (the total number of different species) and evenness (the relative abundance distribution of those species) within a single, individual sample. Beta-diversity measures the compositional dissimilarity and structural variation in microbial communities between different individual samples or clinical groups. N/A: Not Applicable (indicates that the specific parameters or clinical correlates are not relevant or measurable for that specific control/healthy study group).

**Table 2:** Microbiome-Targeted Interventions in HIV: Mechanisms and Evidence.

Intervention Category	Specific Approaches	Proposed Mechanisms	Key Findings in HIV	Current Evidence Level	Key References
Probiotics	*Lactobacillus* spp., *Bifidobacterium* spp.	Direct introduction of beneficial strains; competitive exclusion of pathobionts; enhancement of barrier function	Improved microbial diversity; reduced microbial translocation markers; modest CD4^+^ increases in some studies	Small RCTs show mixed results; effects on inflammation variable	[22,23,65]
Prebiotics	Inulin, FOS, GOS, high-fiber diets	Selective stimulation of beneficial commensals; increased SCFA production; support of endogenous microbiota	Enhanced butyrate production; improved gut barrier markers; reduced immune activation in some cohorts	Limited RCT data; dose and duration effects unclear	[23,45,66]
Synbiotics	Probiotic + prebiotic combinations	Synergistic effects: both introducing and supporting beneficial microbes	Greater microbial richness vs. probiotics alone; enhanced SCFA production; reduced inflammation	Early-phase trials; superior to monotherapy in small studies	[67]
Fecal Microbiota Transplantation (FMT)	Donor stool transplantation	Restoration of microbial diversity; reintroduction of keystone taxa; ecological reset	Increased bacterial richness; partial restoration of butyrate producers; variable immune effects	Pilot studies demonstrate feasibility; safety concerns in immunocompromised	[68,70,74]
Dietary Interventions	High-fiber, Mediterranean, or specific	Modulation of microbial composition	Enrichment of SCFA-producing taxa; reduced	Observational data strong; interventional trials limited	[23,45]
	nutritional regimens	via microbiota-accessible carbohydrates (MACs)	systemic inflammation; improved metabolic parameters		
Engineered Biotherapeutics	Engineered *E. coli*, *Lactococcus*; synthetic biology approaches	Targeted delivery of anti-inflammatory cytokines (IL-10); immune checkpoint modulation; latency reversal	Preclinical proof-of-concept; anti-inflammatory effects in animal models; not yet tested in HIV clinical trials	Preclinical stage; promising but experimental	[24,71,72]
Adjunctive with ART	Microbiome modulation + optimized ART	Bidirectional interaction: microbiome affects drug metabolism; ART alters microbiota	ART regimen-specific effects on microbiome; potential for improved drug efficacy/absorption	Emerging field; requires more pharmacomicrobiomics research	[14,54]

## Data Availability

No new data were created or analyzed in this study. Data sharing is not applicable to this article.

## Abbreviations

The following abbreviations are used in this manuscript:

AhR	Aryl Hydrocarbon Receptor
AI	Artificial Intelligence
ART	Antiretroviral Therapy
BBB	Blood–Brain Barrier
CNS	Central Nervous System
FMT	Fecal Microbiota Transplantation
FOS	Fructooligosaccharide
GALT	Gut-Associated Lymphoid Tissue
GOS	Galactooligosaccharide
HAND	HIV-Associated Neurocognitive Disorder
HDAC	Histone Deacetylase
HIV	Human Immunodeficiency Virus
HLA	Human Leukocyte Antigen
IDO1	Indoleamine 2,3-Dioxygenase 1
I-FABP	Intestinal Fatty Acid-Binding Protein
INRs	Immunological Non-Responders
LAG-3	Lymphocyte-Activation Gene 3
LBP	Lipopolysaccharide-Binding Protein
LPMC	Lamina Propria Mononuclear Cell
LPS	Lipopolysaccharide
LTR	Long Terminal Repeat
MACs	Microbiota-Accessible Carbohydrates
MSM	Men who have Sex with Men
NRTIs	Nucleoside Reverse Transcriptase Inhibitors
PAMPs	Pathogen-Associated Molecular Patterns
PD-1	Programmed Cell Death Protein 1
PLWH	People Living with HIV
RCT	Randomized Controlled Trial
REG3α	Regenerating Islet-Derived Protein 3 alpha
sCD14	Soluble CD14
SCFAs	Short-Chain Fatty Acids
SIV	Simian Immunodeficiency Virus
Th17/Th22	T helper 17/T helper 22 cells
TIM-3	T-cell Immunoglobulin and Mucin-domain containing-3
TLRs	Toll-Like Receptors
TNF-α	Tumor Necrosis Factor-alpha
Treg	Regulatory T cell
ZO-1	Zonula Occludens-1
